# Artistic Prosthesis

**Published:** 1919-07

**Authors:** J. Leon Williams


### ARTISTIC PROSTHESIS

BY J. LEON WILLIAMS, D.D.S., L.D.S.

Somewhere, sometime, away back in the abysmal depths of past time, the animal ancestor of man assumed the erect position, thus freeing his hands for a great variety of new uses. The ever increasing use of the hands, after the upright position had become established, undoubtedly was the most important factor in the evolution of human intelligence and that ascendancy over all other forms of life which the human species rapidly achieved. All of the foremost educational institutions of the world are more and more coming to realize the importance of the use of the hands as an aid and stimulus to brain development and the growth of intelligence. In the interaction of hand and brain and the reaction of the one on the other lies every possibility of future human evolution, and in this future evolution of man those activities which we designate under the general term, art, will play an ever-increasing importance. And the reason for this is that art is the supreme achievement of industry, or, in the words of Prof. Denman Waldo Ross, lecturer on the "Theory of Design" in Harvard University: "Industry means doing things; Art means doing them particularly well."

The future greatness of this country and of every other country in the world depends entirely on the extent to which that statement is appreciated and understood. The future greatness of any profession and of every individual in every profession and calling also depends entirely on the extent to which that great, fundamental truth is appreciated, understood, and carried into effect. The chief trouble with the world today, one might almost say the only trouble, is the lack of a great passion for "doing things particularly well." As one moves up and down this country today and comes in contact with a great variety of activities, one can not help being convinced that the ruling question with many is "how cheaply can I do this work and 'put it across' and get my money for it," and the only part which art plays in such work is the art of making a thing *appear* to be that which it is not, of "dolling up" a thoroughly inferior product until it has the superficial appearance of being done particularly well.

Because these fundamental things apply in dentistry just as they do to any other calling, I want to record some reflections and words of appreciation, as well as some criticisms, of a paper published in the April *Dental Summary* by Dr. F. H. Orton. Because Dr. Orton stands as one of the few who are always urging a realization of Prof. Ross' definition of the difference between industry and art, I am going to dwell a little more at length on these fundamental things in this review of his paper.

When fundamentals are clearly stated and accepted the way is open for the avoidance of mistakes and advance in the right direction.

Every normal human being desires to be successful and happy. I deliberately associate these two conditions of life because happiness is the direct and inevitable result of success, when that term is rightly understood and realized. Let me, then, present a definition of success of which happiness is the natural result.

SUCCESS: *The fullest possible development and expression of all those powers and faculties in the individual which contribute to his own real welfare and the welfare of humanity.*

That definition, you will see, covers not only all possible individual achievement, but the relation of that achievement to all other individuals. It holds the sum and content of all ethics. And in that activity and in that relation lies all human success and the source of all happiness. No individual can be completely successful or completely happy when he is not giving expression to the best that is in him, and *no man is in ethical relationship to his fellows when he is doing less than his best.*

If these propositions are true, it is a matter of vital importance for the individual to seek "the fullest possible development and expression of all those forms which contribute to his own real welfare and the welfare of humanity." There is just one way, one way only, in which these great things of success and happiness can be fully achieved and realized. It is a single and simple rule of conduct. *Let no task or piece of work pass from your hands until you have performed the task, until you have finished that work to the best of your ability.*

The reactionary effects on human development of that rule of life in one's work is beyond all computation. The deep satisfaction of work well done is in itself one of the chief sources of human happiness. And the reactionary effects of this sort of work are not confined to our mental, moral or spiritual faculties. It also profoundly affects our physical bodily functions and qualities.

The state of mental exaltation growing out of the consciousness that we have done our level best is one of the greatest promoters of health and general physical well-being.

To begin each day's work with the intention and firm determination to do that work a little better than you have ever done it before, is to put every nerve fibre of the body and every nerve cell of the brain in the path of development and progress. And there is no other way of accomplishing this great end. In the measure of the exercise of power is to be found the index of its increase. The fatal mistake that the vast majority make is in estimating their own powers too cheaply, in always holding the thought that they can not reach the heights that others have won. Individuals vary in their capacity for development, but there is no normal human being who may not profoundly astonish himself at the progress he has made in a single year by laying out all his time in a systematic manner—so much for the regular day's work, so much for reading and study, and so much for recreation, and then doing everything connected with these several groups of activities to the fullest reach of his ability. He will have no need to ask any one to show him the way to success and happiness—he will have found the royal road.

To do one's work in the best possible manner is to constantly exalt it, and to exalt one's work is to exalt one's self. Drudgery is largely a mental attitude, a debasing, degrading mental attitude. It can be banished by a constant study of how to do the simplest things with the greatest possible art. It is, of course, perfectly easy to present instances in ridicule of these statements. But nothing has ever escaped the ridicule of those who find greater delight in tearing down than in building up, more satisfaction in discouraging than in encouraging their fellows.

In this critical time of the world's history we frequently hear it said of men who are trying to lay the foundations for a better civilization that they are idealists. And the term is used in reproach. The only comment called for is that God alone knows if there would be any sort of a civilization on earth today if there had not been great idealists in the past who endured the same taunts that blind, faithless reactionism is flinging out today.

Let me, then, emphasize this fact: If the experience of nearly fifty years of professional work has taught me anything, it has demonstrated that the only path of development and progress is that in which the highest possible perfection of work is aimed at, and in which the greatest possible effort is made to reach the ideal formed in the mind as the aim. And I can not attribute a single failure of my own to any other cause than non-observance of these principles.

These are some of the reflections that followed the reading of Dr. Orton's stimulating and thought-provoking paper, and now let me come to some of the details of this paper which seem to me to call for comment and criticism.

On going over this article carefully I find the following salient points to which I wish to call attention:

(1) *Definition of art in relation to dental prosthesis.*
(2) *To what extent should the manufacturer be expected to supply teeth made in the most exact imitation of nature that it is possible to produce?*
(3) *On the nature of color in natural teeth and its imitation in artificial teeth.*
(4) *On the standardizing of the color of teeth by the classification of people into types and classes.*
(5) *On the modification of color in artificial teeth by surface markings.*
(6) *Apparent changes in color due to contrast and nerve exhaustion.*

(1) *Definition of art in relation to dental prosthesis.*—Dr. Orton finds the most appropriate definition in the old epigram: "True art is to conceal art." But he is careful to state that this definition applies especially to crown and bridgework and that it expresses the limitations of imitative art. In most cases of crown and bridgework we have to match natural teeth with artificial ones as perfectly as we can. We have to copy all the peculiarities, defects, and so-called "accidents of nature" to the best of our ability. But we should never lose sight of the fact that imitative art is not considered a high form of art, and that the moment we find ourselves free from the arbitrary demands of nature's imperfections we are bound to endeavor to utilize the principles of a more exact science and a higher art. When we are forced to match natural teeth we may find ourselves obliged to conform to various defects and disharmonies of nature. We may have to insert a tooth of the tapering or second-class form in a square face, or a square tooth of the first class in an ovoid face. We are bound to the wheel of nature's defects and mistakes, we are confined to a purely imitative art and can not help ourselves. But when the natural teeth are all lost we are free to follow the teaching of a scientific classification and the principles of a higher form of art than that of mere imitation—the art in which harmony of tooth form to face form is the first essential. It can not be too often repeated that nature rarely achieves perfect harmony in any of her work. As Ernest Garett in his recent very able work on "Art Principles" says: "Nature never produces a perfect form without a perfect countenance . . . but it is within the power of art to correct the work of nature in these respects."

And again: "In the case of a fully developed animal when the position of parts is fixed, a type may be conceived which is superior in symmetry and harmony to any individual of the species produced by nature."

It is the duty of the dentist to make himself fully acquainted with the principles of harmony which exist between tooth form and face form and to give his patients the benefit of his knowledge. He will find the study leading to this knowledge most fascinating, and in the application of this higher form of art in dental prosthesis he will experience an ever-increasing delight to himself and an ever-growing esteem and appreciation on the part of his patients. One of the great rewards of doing the best that we can in our work is an ever-growing interest in and love of the work itself.

(2) *To what extent should the manufacturer be expected to supply teeth made in the most exact imitation of nature that it is possible to produce?*—Dr. Orton, in considering this phase of his subject says: "I believe that we have a right to demand that the manufacturer furnish us with teeth which show all the peculiarities of age." And he further specifies some of the peculiarities such as "abrasion, erosion, and even atrophied enamel formation," which I infer he would have the manufacturer imitate in the teeth which he offers the profession.

Let us see just how this proposition would work out practically. Dr. Orton very truly says that we should go to nature and study her very carefully. We can not do too much of that, for it is the basis of all art as of all other forms of knowledge. Let us suppose, then, that the manufacturer makes a most exhaustive study of, I will not say all of the defects and peculiarities of natural teeth, for that would obviously be impossible, but of a wide range of these "accidents of nature" in form, markings and stainings.

The number of these markings is, as I have intimated, infinite or indefinite. They are not alike in any two mouths. They vary in the individual teeth in any given mouth. The stock required to produce any considerable fraction of the vast number of these variations as found in nature would be far beyond the capacity of any retail dealer to handle. But if such a stock could be accumulated it is highly probable that a dentist might spend a whole day in going over such a stock without finding a suitable match for the defects, special markings, stainings or other peculiarities of the case in hand.

A limited number of sets of teeth of this character might be supplied for full cases, but obviously, it would be completely impracticable for the manufacturer to attempt to produce a stock of such teeth for crowns, bridgework facings, or partial cases of platework where natural teeth have to be matched. In all work of this sort the utmost that the manufacturer can do is to supply standard forms conforming to types and the most common modifications of types, in suitable colors. On this foundation the dental artist must produce the particular effects he desires for any given individual case.

(3) *On the nature of color in natural teeth and its imitation in artificial teeth.*—In the last analysis all color is due to structure, to the molecular arrangement of matter. But our perception of color as it appears, for illustration, in the great variety of tints seen in the clouds of an evening sky, and a similar variety of tints or hues observed in fabrics displayed in a shop window is due to widely different conditions. In the first instance our perception of color is due to phenomena closely akin to spectrum analysis; in the second to pigments which absorb certain colored rays of light and reflect others.

These two exhibitions of color phenomena have a direct bearing on the problem of producing color in artificial teeth in imitation of natural teeth. Color in porcelain teeth must be produced by mineral pigments.

The appearance of color in natural teeth is probably due to physiological and structural conditions in which pigmentary deposits play little or no part.

In a paper which I read some six years ago before the First District Dental Society of New York, I called attention to the Lovibond method of color analysis and its possible application to the determination of color in natural teeth. Subsequently Dr. G. W. Clapp went to England and spent some time in the Lovibond Laboratory, where with the assistance of experts the colors in several sets of teeth were analyzed. But as this method of color analysis is akin to that of spectrum analysis, and as the primary colors of the spectrum can not be used as the basis of coloration with pigments, the knowledge gained by this system of analysis does not get us forward much. And the reason is, as I have intimated, that when we begin experimenting in the coloration of porcelain we are unable to make use of those colors and tints which we used in our methods of analysis. We have to gain a new kind of knowledge which is quite independent of that obtained by our methods of analyzing.

I am in complete agreement with Dr. Orton that the methods at present in use in the coloration of artificial teeth should be revolutionized, and I have no doubt that effects in much closer resemblance to nature can and will soon be obtained and that the scale will be **far simpler** than that now in use.

I am inclined to agree with Dr. Orton as to the principal basis of color in natural teeth, but I have to admit that our present opinions are largely the result of scientific speculation.

A large amount of experimental work must be conducted in this field before we shall find ourselves on solid ground.

(4) *On the standardizing of the color of teeth by the classification of people into types and classes.*—I believe this can be done and that it will be done in the near future. And I believe that the possibility for advance in the improvement in the colors of artificial teeth, based on the ascertained colors of natural teeth described by scientific nomenclature, are as great as were the possibilities for improvement in form ten years ago. I have said that scientific methods of color analysis can help us but very little in perfecting a color scheme for artificial teeth, because that color scheme must be perfected entirely by experimental methods which have scarcely anything in common with the scientific procedure of color analysis. But before we can know what we want for color in artificial teeth we must first know that the colors are in natural teeth, and I have no doubt these essential primary facts can be ascertained by systems of color analysis now in use. These facts once in hand there must follow a long series of experiments with the few mineral colors available in the effort to match the colors we have found in the natural teeth. The crux of this whole difficult problem lies in the perfecting of a set of color symbols which will at once and at the same time stand for the color of natural teeth as expressed by the results of a system of color analysis, analogous to spectrum analysis, and the results of a purely empirical or experimental method with mineral pigments. This is not an easy matter, but I believe the thing can be done. It must be done if we are to achieve entirely satisfactory results, and I hope Dr. Orton will be able to work out a set of symbols which will help us to accomplish this great advance.

(5) *On the modification of color in artificial teeth by surface markings.*—I have said that in the last analysis our perception of color is due to the molecular structure of the object reflecting the color. But this color is somewhat modified by the nature of the surface. The more highly polished the surface the stronger the tendency for the color to pass into higher or lighter tints and for surrounding colors to be reflected from this highly polished surface.

But the interior structure and the surface of a natural tooth can only be perfectly seen and thoroughly understood by the aid of the microscope. This microscopic structural condition influences the color as we see it. But it is quite impossible to imitate this interior or surface structure in porcelain. We therefore have to call art to our aid and endeavor, by various means, to produce such a surface on our porcelain tooth as will give the same color and structural *appearance,* to the eye, at a little distance, as we see in the natural tooth. The polished surface of the natural tooth must be modified in some way so that the rays of light will be dispersed and the purely reflecting surface obliterated.

(6) *Apparent changes in color due to contrast and nerve exhaustion.*—Dr. Orton has treated this phase of his subject in a very thorough and scientific manner, and the only added suggestion I have to make is that the dentist, when engaged in matching the color of teeth, should have close at hand some bright blue object to which his eyes may be turned every few moments to neutralize the color exhaustion effects of prolonged gazing at the yellow of the teeth.

The best work treating this subject that I have found is, "Color in Everyday Life," by Prof. Louis Weinberg, and published by Moffit, Yard and Co., of New York.

If every dentist who reads this would order that book (price $2.50) I am sure he will thank me many times for calling his attention to it. Art enters into our work to such an extent, as Dr. Orton has so forcibly pointed out, that every dentist should make himself familiar with the general principles and practical application of all art matters that concerns his work.

Philip Gibbs, the famous war correspondent who is returning to England after a two months' lecturing tour in this country, has just published his farewell message to the American people in the New York *Times*. It is an arresting and noteworthy communication. He tells us that he is convinced we are at heart a people with high ideals. And he tells us that the hour of our destiny has struck, that our supreme moment is at hand, that we are summoned "to the highest place in the world's democracies." I believe this to be an utterance of the most momentous truth we have ever faced. And in this great privilege of holding up ideals to the world each and every one of us has his opportunity and what should be his willing and glad duty. American dentistry has, during the war just ended, added much to the high reputation it has long held. There is greater work for the future. The standard of any profession is determined not by the work of a few, but by the general level of accomplishments and ideals of the majority.

Dentistry, because its demands enforce an intimate relation between the work of the hands and the action of the brain, is an occupation that should greatly foster individual development. And it will always do this if high ideals are constantly kept before the mind.

Dr. Orton quotes the remark of a prominent manufacturer of artificial teeth who said: "When the dental profession knows what it wants, we will supply the demand.'' I do not know who said that, but I do wish that the words could be printed in letters large enough to be read at a distance of a hundred feet and hung up in every place where dental organizations meet. The dental manufacturer may never be entirely actuated by philanthropic motives (how many professional men are?) but he is wise enough to know that his success as a business man depends upon his keeping abreast of concerted demand.

In the past the dental profession has sometimes been *led* by the manufacturer. Let us hope that time is permanently in the past.

The great new idea, which is but the revival of a very old idea, now at the front in the minds of all progressive men is co-operation—working together, all working for the benefit of all.

The leading dental manufacturers stand ready to cooperate with the profession in all endeavors to realize the highest ideals.

And there is no way by means of which we can realize our ideals in many directions except by intelligent cooperation with the manufacturer. Dr. Orton has pointed out an advanced step which should be taken immediately. If he is supported in his demands by any considerable body of men in the profession his demands will be met, and the work will be done—*The Dental Digest* for June, 1919.

